# Didactic adaptation of basketball in physical education to reduce anxiety and emotional eating in adolescent girls with obesity: a randomized controlled trial

**DOI:** 10.3389/fpsyg.2026.1753266

**Published:** 2026-04-13

**Authors:** Oumayma Slimi, Amayra Tannoubi, Raouf Nasri, Mohamed Abdelkader Souissi, Santo Marsigliante, Antonella Muscella, Jolita Vveinhardt

**Affiliations:** 1 High Institute of Sport and Physical Education, University of Sfax, Sfax, Tunisia; 2 Research Laboratory: “Education, Motricité, Sport et Santé”, EM2S, LR19JS01, High Institute of Sport and Physical Education of Sfax, University of Sfax, Sfax, Tunisia; 3 Department of Education, High Institute of Sport, and Physical Education of Gafsa, University of Gafsa, Gafsa, Tunisia; 4 Sports Performance Optimization Research Laboratory (LR09SEP01), National Center for Sports Medicine and Science (CNMSS), Tunis, Tunisia; 5 Physical Activity, Sport and Health Research Unit, UR18JS01, National Observatory of Sport, Tunis, Tunisia; 6 Department of Biological and Environmental Sciences and Technologies (Di.S.Te.B.A.), University of Salento, Lecce, Italy; 7 Klaipėdos valstybinė kolegija/Higher Education Institution, Klaipėda, Lithuania

**Keywords:** adapted physical education, adolescent girls, basketball training, emotional eating, motor performance, obesity, sport-related anxiety

## Abstract

**Background/objectives:**

Adolescent girls with obesity often experience elevated sport-related anxiety and emotional eating, which impair both physical and psychological health. This study aimed to evaluate the effects of a didactically adapted basketball program on body mass index (BMI), emotional eating, sport-related anxiety, and technical performance in adolescent girls with moderate obesity.

**Methods:**

In this two-arm, parallel-group randomized controlled trial, 64 participants aged 15–16 years were randomly assigned to an experimental group (EXP, *n* = 32), receiving a 7-week adapted intervention, or a control group (CONT, *n* = 32), following standard physical education sessions. Assessments included BMI, the Dutch Eating Behavior Questionnaire (DEBQ), the Sport Anxiety Scale-2 (SAS-2), and standardized passing and shooting drills, administered pre- and post-intervention under supervised and confidential conditions.

**Results:**

The adapted program led to significant improvements in BMI (*p* < 0.001, *d* = 0.65), emotional eating (*p* = 0.0003, *d* = 0.74), and sport-related anxiety (*p* < 0.001, *d* = 0.95), as well as enhanced passing and shooting accuracy (*p* = 0.004 to < 0.001, *d* = 0.56–0.72). Reductions in anxiety and emotional eating were significantly correlated with gains in technical performance (*r* = −0.31 to −0.41, *p* < 0.01).

**Conclusions:**

Pedagogically adapted basketball sessions represent an effective and inclusive approach to enhance motor skills, regulate emotions, and improve body composition in adolescent girls with moderate obesity. These findings emphasize the value of progressive, emotionally supportive, and socially engaging physical education programs tailored to vulnerable adolescent populations.

## 1 Introduction

Childhood and adolescent obesity has become a major public health concern worldwide (Nilson et al., 2025; Lister et al., 2023), affecting populations in both developed and low- to middle-income countries (Jebeile et al., 2022; Lister et al., 2023), with particularly high prevalence in urban areas (Lister et al., 2023; Restrepo, 2022). Among adolescents, obesity is especially common in girls (Manoussi et al., 2025; Yao et al., 2025). This makes this group particularly vulnerable to both physical and psychological health risks (Insaf et al., 2024; Trübswasser et al., 2021; Eker et al., 2017). Adolescent girls with obesity face multiple health risks, including cardiovascular disorders (Raj, 2012), hypertension (Abbas et al., 2025; Tran et al., 2025), metabolic syndrome (Anton-Pǎduraru et al., 2025; Guijarro de Armas et al., 2012; Münte et al., 2025), and type 2 diabetes (Luk et al., 2025). Beyond physical health, obesity is associated with significant psychological challenges (Putra et al., 2025), such as increased anxiety (Celiloglu et al., 2025; Zhuang et al., 2025), low self-esteem (Fore-Williams and Derouin, 2025), body dissatisfaction (Enver et al., 2025; Jebero et al., 2025), and emotional eating behaviors (Cuyan-Zumaeta et al., 2025; Demir Kösem and Bektaş, 2025). These combined physical and psychological consequences often persist into adulthood (Putri et al., 2025; Inge et al., 2013), emphasizing the need for early and targeted interventions (Slimi et al., 2023, 2025b). Targeting this population with adapted basketball sessions may not only improve their physical and psychological health but also foster social inclusion, teamwork, confidence in school settings, and positive engagement in physical education, highlighting the broader educational and social significance of the intervention.

Obesity generally results from an excessive accumulation of adipose tissue, often due to unhealthy eating habits and sedentary behaviors (Agrawal et al., 2013; Beltrán-Carrillo et al., 2022; Nurwanti et al., 2018; Intorre et al., 2025). Emotional eating, defined as the tendency to consume food in response to emotions such as stress, anxiety, or frustration rather than physiological hunger (Demir Kösem and Bektaş, 2025; Shriver et al., 2020; Dakanalis et al., 2023; van Strien, 2018). This behavior plays a central role in the development and maintenance of obesity among adolescent girls. Emotional eating has been strongly linked to heightened anxiety and is considered a key factor contributing to sustained weight gain during adolescence (Kidwell et al., 2024; Dakanalis et al., 2023; Shriver et al., 2020; Wardani et al., 2024). Psychological stress and maladaptive eating behaviors interact in a reciprocal manner. This interaction may create a cycle that reinforces both obesity and emotional distress (Demir Kösem and Bektaş, 2025; Dakanalis et al., 2023; Torres and Nowson, 2007; Esen öksüzoglu et al., 2024). Engaging in structured physical activity, particularly in supportive sports environments, may help regulate emotional eating by providing alternative coping strategies, improving mood, and fostering a sense of mastery and self-efficacy.

Anxiety, characterized by heightened tension and worry in response to perceived stressors (Gori et al., 2023), can be particularly pronounced in the school context, especially during physical education (PE) sessions (Ligon et al., 2025; Yang and Sun, 2025; Yous et al., 2025; Chen et al., 2024; Aydi et al., 2025). Basketball was specifically chosen as the intervention tool because it is a popular team sport in school settings, requiring coordination, agility, and teamwork, while allowing pedagogical adaptations to meet the physical and psychological needs of adolescents with obesity. Adolescent girls with obesity often experience discomfort, low self-confidence, and feelings of incompetence when participating in physical activities, especially in team sports like basketball, which demand coordination, speed, and endurance (Slimi et al., 2025a, 2024; Noonan, 2022). Traditional basketball sessions are widely used in physical education. However, they are often insufficiently adapted to the abilities of students with obesity. This lack of adaptation may exacerbate feelings of inadequacy and social marginalization (Slimi et al., 2025b; Noonan, 2022). This situation may inadvertently increase emotional eating and reinforce negative psychological states, creating a cycle of anxiety and unhealthy eating patterns.

Adolescent girls with obesity typically engage in lower levels of physical activity, limiting their opportunities to develop motor skills and participate fully in school-based sports (Alexatou et al., 2025). These limitations, coupled with anxiety and emotional eating, contribute to a decline in overall physical and psychological wellbeing (Braden et al., 2018). Physical education classes, when not adapted, may unintentionally highlight differences in ability, resulting in frustration, withdrawal, and social exclusion for students with obesity (Trout and Graber, 2009).

Despite growing awareness of the challenges faced by girls with obesity in physical education (PE) settings, few studies have proposed and tested practical approaches for adapting team sports such as basketball to their specific psychological and physical needs. Previous intervention studies in school-based physical activity programs have reported beneficial effects on weight management, psychological wellbeing, and physical fitness among adolescents with obesity. However, most of these interventions have primarily focused on individual or fitness-based activities, such as aerobic exercise programs, circuit training, or general physical activity promotion. In contrast, relatively few studies have investigated the potential benefits of didactically adapted team sports implemented within physical education settings. Therefore, the present study aimed to evaluate the effects of didactic adaptations of basketball sessions implemented in PE. Specifically, the study examined their effects on sport-related anxiety, emotional eating behaviors, motor learning outcomes, and BMI among adolescent girls with moderate obesity. This approach was designed to provide a feasible, evidence-based strategy to reduce emotional eating and anxiety, enhance motor competence, and promote both social inclusion and physical health in this vulnerable population.

## 2 Materials and methods

### 2.1 Participants and randomization

A total of 74 adolescent girls enrolled in the first year of secondary school in Sidi Bouzid, Tunisia, were systematically recruited from eligible volunteers. They were drawn from eight different classes. Of these, eight participants were excluded—five for not meeting the BMI criteria for obesity and three for lacking parental consent. The remaining 66 participants were randomly assigned to either the experimental group (EXP, *n* = 33) or the control group (CONT, *n* = 33). All participants began the intervention, but two were lost to follow-up (one per group due to illness), leaving 64 participants (32 per group) included in the final analysis. No data was excluded due to protocol deviations.

**Figure 1:**
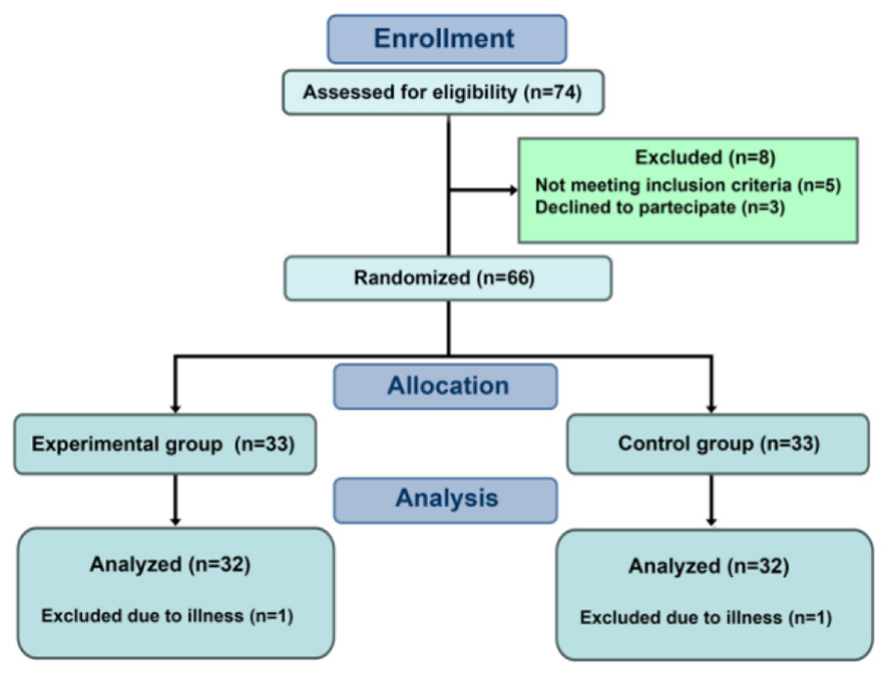
CONSORT flow diagram.

Participant selection specifically targeted adolescents meeting BMI criteria for obesity, determined according to age- and sex-specific percentiles defined by World Health Organization (WHO) guidelines. Exclusion criteria included the absence of signed parental consent, lack of commitment to participate throughout the study, incomplete parental questionnaires, inability to perform physical tests, or the presence of musculoskeletal, neurological, respiratory, or mental health disorders. These conditions were verified through a parent-completed medical history questionnaire and a basic physical examination conducted by a healthcare professional (Figure 1).

Participants (aged 15–16 years) were randomly assigned by the principal investigator (O.S., co-author) using a computer-generated randomization list in SPSS (version 24) to either the EXP or CONT group. Following randomization, no significant between-group differences were observed in mean weight, height, or BMI percentiles (*p* > 0.05, ANOVA), ensuring baseline equivalence (Table 1).

**Table 1:** Anthropometric measurements.

Groups	EXP (*n* = 32)	CONT (*n* = 32)
Age (years)	15.4 ± 0.3	15.5 ± 0.4
Height (cm)	167.1 ± 5.2	166.8 ± 5.1
Weight (kg)	87.5 ± 6.5	87.9 ± 6.0
IMC(kg.m^−2^)	31.86 ± 1.1	32.07 ± 1.32

Allocation concealment was ensured by blinding the principal investigator to participant identities during the generation of the randomization sequence, and group assignments were placed in sealed opaque envelopes, opened only immediately before the first intervention session.

All participants and their parents or legal guardians were fully informed about the study's objectives, procedures, potential benefits, and risks. Written parental consent and participant assent were obtained before the start of the study. Ethical measures were taken to respect participants' sensitivity regarding their physical appearance and to minimize discomfort during all procedures. The study protocol complied with the ethical principles of the Declaration of Ndebele (2013) and was approved by the Ethics Committee of the Faculty of Medicine of Sfax (Approval No. 048/2022). Continuous follow-up procedures were implemented to ensure participant comfort, including periodic check-ins and opportunities to report any discomfort during assessments, with trained staff available to help as needed.

Based on an *a priori* power analysis conducted using G^*^Power 3.1.9.4 (University of Düsseldorf, Germany), a sample size of 64 participants (32 per group) was determined to be sufficient to detect a medium effect size (Cohen's *f* = 0.25) for a mixed-model ANOVA with two groups and two time points (Group × Time interaction), assuming α = 0.05 and statistical power (1 – β) = 0.80.Data are presented as mean ± standard deviation. EXP, Experimental group; CONT, Control Group; IMC, BMI, body mass index.

### 2.2 Experimental procedure

This two-arm, parallel-group randomized controlled trial, conducted in accordance with CONSORT guidelines, aimed to evaluate the effects of didactic adaptations of basketball sessions in physical education on anxiety, emotional eating, basketball performance, and BMI in adolescent girls with obesity. One week before the intervention, participants were familiarized with testing procedures and equipment. Assessments of basketball performance (passing, dribbling, free throws) and BMI were conducted before (T0) and after the 7-week intervention (T1), while anxiety and emotional eating were evaluated after the first and last sessions. Testing occurred during physical education classes (9:00–11:00 a.m.) to ensure consistency. During the testing sessions (pre- and post-intervention assessments), no verbal feedback was provided to participants to ensure that motor performance and psychological measures were not influenced by external cues.

The intervention consisted of two 1-h sessions per week for 7 weeks, each session systematically structured into a 15-min standardized warm-up, a 40-min main phase, and a 5-min standardized cool-down. The warm-up and cool-down activities were identical for all participants to ensure baseline consistency and reproducibility. The main phase consisted of predetermined drills, which were uniformly applied to all participants within each group: the control group followed traditional drills, whereas the experimental group followed the same didactically adapted drills for all participants, ensuring that every adolescent received an identical adapted program. This approach maintained session uniformity while systematically tailoring the intervention to the specific physical and psychological needs of the participants. All sessions were conducted by the same instructors to ensure consistency of delivery and minimize instructor-related variability.

To control for external factors that could affect sleep quality and subsequent motor and cognitive performance, participants were instructed to stop exposure to blue light from screens and electronic devices at least 3 h before their usual bedtime throughout the study (Souissi et al., 2025; Dridi et al., 2026).

### 2.3 Anthropometric measurements

Methods Height and weight were measured using a stadiometer and an electronic scale (Tanita, Tokyo, Japan), with measurements recorded to the nearest 0.01 m for height and 0.01 kg for weight. All assessments were conducted by the same operator between 9:00 and 11:00 a.m. to ensure precision and consistency. Body Mass Index (BMI) was calculated as weight (kg)/height^2^ (m^2^). Participants' weight status was classified using age- and sex-specific BMI percentiles according to WHO guidelines: above the 95th percentile indicated obesity, and above the 85th percentile indicated overweight. To minimize discomfort among participants sensitive to body image, the measurements were presented as physical fitness assessments, allowing participants to learn their BMI in a discreet and respectful manner.

### 2.4 Intervention program

#### 2.4.1 Adapted intervention

The experimental program consisted of a 7-week basketball training intervention, comprising two 60-min sessions per week, and was progressively structured to address the needs of adolescent girls with obesity while evaluating the effects of didactic adaptations on anxiety, emotional eating, basketball performance, and BMI, consistent with prior research indicating that significant physical and psychological changes can occur over a similar duration (Zhuang et al., 2025).

During week 1, sessions focused on familiarization with basketball through the introduction of basic rules and fundamental skills. In the adapted program, activities alternated between high-intensity exercises and active recovery, minimizing competitiveness to foster engagement within a supportive, low-pressure environment.

In weeks 2 and 3, 1-on-1 drills were introduced. For the adapted sessions, defensive pressure was reduced by allowing defenders to use only one hand and to engage only after the attacker-initiated movement, thus facilitating participation and confidence among overweight students.

In weeks 4 and 5, 3-on-3 drills were adjusted to a 3-attackers-vs.-2-defenders format, increasing offensive opportunities and encouraging inclusion in team play.

Finally, in weeks 6 and 7, full 5-on-5 games were implemented. In the adapted version, mandatory substitutions occurred at each offensive transition, ensuring frequent rest, reducing fatigue, and promoting both psychological comfort and active engagement in learning.

Overall, these didactic adaptations were designed to enhance enjoyment, reduce anxiety, support emotional regulation during physical activity, and optimize basketball performance, while contributing to improvements in BMI and overall wellbeing.

#### 2.4.2 Control group

The control group followed the regular physical education basketball curriculum delivered at the school. Sessions included standard technical drills (passing, dribbling, and shooting) and small-sided games conducted according to the usual pedagogical practices of the teacher.

Unlike the experimental group, no didactic adaptations (e.g., reduced defensive constraints, mandatory rotations, or progressive task structuring specifically designed for students with obesity) were implemented.

### 2.5 Psychological measures

#### 2.5.1 Emotional eating

Emotional eating behavior was assessed using the “Emotional Eating” subscale of the Dutch Eating Behavior Questionnaire (DEBQ; van Strien, 2018), in its French validated version by Lluch et al. (1996). This subscale consists of 13 items; each rated on a five-point Likert scale (from “never” to “very often”). A global score was obtained by averaging responses across the 13 items, with higher scores indicating a stronger tendency to eat in response to negative emotions. The internal consistency of the subscale in our sample was excellent (Cronbach's α = 0.92).

Questionnaires were self-administered independently by participants, with standardized instructions provided. Trained staff were available only to clarify wording or formatting when necessary, without influencing participant responses.

#### 2.5.2 Sport-related anxiety

Sport-related anxiety was assessed using the “Cognitive and Somatic Anxiety” subscale of the French version of the Sport Anxiety Scale-2 (SAS-2; Smith et al., 2006), in its French validated version by Martinent et al. (2010). This subscale includes 10 items; each rated on a four-point Likert scale (from “not at all” to “very much”). A global score was calculated by summing the responses, with higher scores reflecting greater levels of sport-related anxiety. The internal consistency in our sample of adolescent girls with obesity was good (Cronbach's α = 0.87).

Before the first administration, a pilot test was conducted to familiarize participants with the questionnaires and identify any issues related to wording or formatting. Based on participant feedback, minor adjustments were made to enhance clarity and cultural relevance. Questionnaires were administered in group settings at the end of physical education sessions, following standardized instructions provided by the researchers. Teaching staff were available to clarify any questions and ensure full comprehension. Participants were given approximately 15–20 min to complete the questionnaires. All responses were anonymized prior to analysis, and confidentiality was strictly maintained. Only the research analyst (O.S.) had access to the raw data. Final mean scores were calculated and used for subsequent statistical analyses.

### 2.6 Basketball athletic performance evaluation

During weeks six and seven, which included four sessions of 5-on-5 basketball games (two sessions per week), students who were resting, recovering, or not actively participating were progressively trained in the use of the observation grid. This training ensured that, by the time of the final evaluation, they were able to reliably record peer performance. The training included practice sessions, calibration exercises, and demonstration of scoring procedures to minimize inter-rater variability and enhance consistency.

At the end of the intervention, individual progress was assessed through a combined self-assessment and peer-evaluation approach designed to develop students' capacity to critically appraise their own performance and provide constructive feedback to peers. The reliability of peer- and self-assessments was quantified using Cronbach's alpha, and inter-rater reliability between observers was calculated using intraclass correlation coefficients (ICCs), demonstrating high internal consistency (Cronbach's α ≥ 0.88) and inter-rater reliability (ICC ≥ 0.85).

To assess both baseline abilities and progress following the adapted basketball program, performance was evaluated at two distinct points: a pre-test (T0) and a post-test (T1). At T0, the initial evaluation was conducted exclusively by the teacher to ensure measurement reliability in a context where students were novices and unfamiliar with the observation grid. Students completed standardized individual technical drills comprising 10 passes and 10 shots, allowing measurement of core performance indicators. At T1, at the conclusion of the program, each student's performance was evaluated during a 7-min 5-on-5 basketball match serving as the end-of-cycle test. All participants were aware that their performance was being assessed; however, to minimize potential bias due to this awareness, all testing sessions were conducted under strictly standardized conditions, and no verbal feedback or guidance was provided during the evaluations. Observers followed a standardized observation protocol to ensure objectivity and procedural consistency. Two trained observers supervised each match: one recorded quantitative data while the other monitored procedural adherence, ensuring that scoring reflected true performance without influence.

This evaluation employed a mixed approach combining peer observation and self-assessment, made possible by prior training in the use of the observation grid. The same performance indicators as in T0 were recorded to ensure reliable comparison between the two assessments. Two trained observers were assigned specific roles during each match, with one recording quantitative data in the observation grid and monitoring procedural adherence. Collected data were communicated to the respective teams during timeouts and at the conclusion of the match. These measurements were subsequently used for statistical analyses to examine the effects of the adapted basketball program and to identify group- and gender-specific differences in passing and shooting performance.

### 2.7 Statistical analysis

All statistical procedures were performed using Statistica software (version 10.0; StatSoft Inc., Krakow, Poland). Prior to hypothesis testing, the Shapiro–Wilk test was applied to verify the normal distribution of the data, and the Levene's test confirmed the homogeneity of variances across groups. These preliminary checks supported the use of parametric analyses.

To examine the effects of the intervention on the main dependent variables—Body Mass Index (BMI), emotional eating (DEBQ), anxiety, and technical basketball performance (passing and shooting)—a two-way mixed-design analysis of variance (ANOVA) was conducted with one between-subjects factor (Group: experimental [EXP] vs. control [CONT]) and one within-subjects factor (Time: pre-test [T0] vs. post-test [T1]). When a significant Group × Time interaction was detected, Bonferroni-adjusted *post hoc* tests were performed to identify the specific pairwise differences within and between groups.

The magnitude of effects was quantified using partial eta squared (η*p*^2^) for ANOVA analyses and interpreted as small (η*p*^2^ ≥ 0.01), medium (η*p*^2^ ≥ 0.06), or large (η*p*^2^ ≥ 0.14), according to conventional benchmarks (Cohen, 1988). For pairwise comparisons, Cohen's *d* effect sizes were calculated and interpreted as small (*d* ≥ 0.2), moderate (*d* ≥ 0.5), or large (*d* ≥ 0.8).

To explore the relationships between psychological and motor improvements, Pearson's product–moment correlation coefficients (*r*) were computed using change scores (Δ = T1 – T0) for each variable. The strength of correlations was interpreted following standard conventions: weak (*r* < 0.3), moderate (0.3 ≤ *r* < 0.5), and strong (*r* ≥ 0.5).

All statistical data are presented as means ± standard deviations (SD). The threshold for statistical significance was set at *p* < 0.05 for all analyses, and *p*-values were adjusted for multiple testing using the Holm–Bonferroni correction where appropriate.

## 3 Results

### 3.1 Body mass index (BMI)

**Figure 2:**
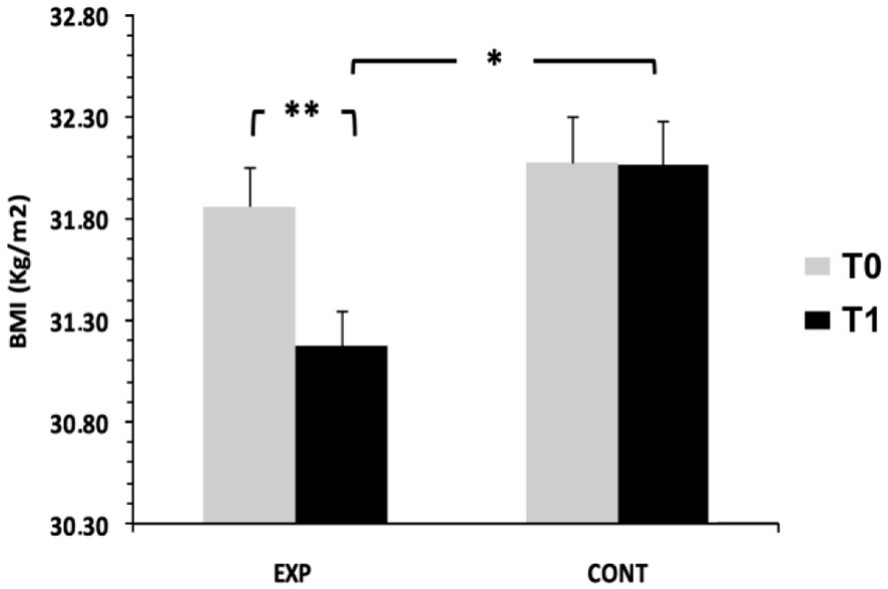
Body Mass Index (BMI) measured before (T0) and after the intervention (T1) in the control (CONT) and experimental (EXP) groups. *p < 0.05; **p < 0.001, Bonferroni post hoc tests.

A two-way mixed ANOVA [2 Groups × 2 Time Points] with repeated measures on the time showed a significant main effect of time, *F*_(1, 62)_ = 20.71, *p* < 0.001, η^2^*p* = 0.25, and a significant group × time interaction, *F*_(1, 62)_ = 19.25, *p* < 0.001, η^2^*p* = 0.24, indicating that BMI reduction differed between groups. The main effect of group did not reach statistical significance, *F*_(1, 62)_ = 3.74, *p* = 0.058, η^2^*p* = 0.06. Bonferroni *post hoc* tests revealed a significant decrease in BMI from pretest to posttest in the experimental group (*p* < 0.001, *d* = 0.65), whereas the control group showed no significant change. Posttest BMI was significantly lower in the experimental group than in controls (*p* < 0.05, *d* = 0.78). Mean BMI decreased from 31.86 ± 1.10 to 31.18 ± 1.01 in the experimental group, while remaining stable in the control group (32.07 ± 1.33 vs. 32.07 ± 1.26; Figure 2).

### 3.2 Emotional eating (DEBQ)

**Figure 3:**
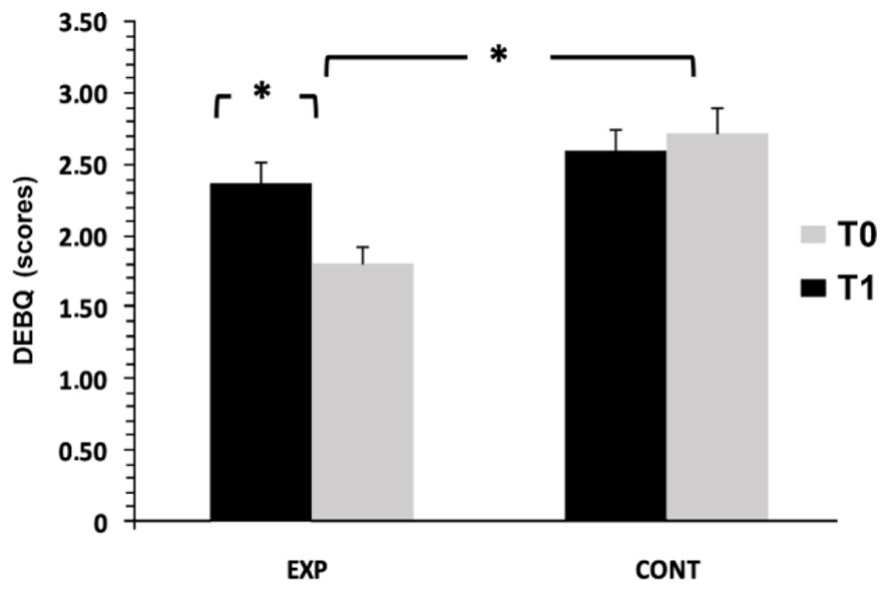
Emotional eating (DEBQ) measured before (T0) and after the intervention (T1) in the control (CONT) and experimental (EXP) groups. *p < 0.001, Bonferroni post hoc tests.

To investigate the evolution of emotional eating behaviors, a mixed two-way ANOVA [2 Groups × 2 Time Points] with repeated measures on the time factor was performed. The analysis revealed significant main effects of group, *F*_(1, 62)_ = 8.50, *p* < 0.01, η^2^*p* = 0.12, and time, *F*_(1, 62)_ = 5.69, *p* = 0.020, η^2^*p* = 0.08, as well as a significant group × time interaction, *F*_(1, 62)_ = 14.05, *p* < 0.001, η^2^*p* = 0.18. *Post hoc* tests showed a significant reduction in emotional eating in the experimental group (*p* < 0.001, *d* = 0.74), while the control group increased slightly (*p* < 0.001, *d* = 0.13). Between-group comparisons at posttest also favored the experimental group. Descriptively, DEBQ scores decreased from 2.38 ± 0.83 to 1.81 ± 0.64 in the experimental group and increased from 2.59 ± 0.87 to 2.72 ± 1.02 in controls (Figure 3).

### 3.3 Anxiety

**Figure 4:**
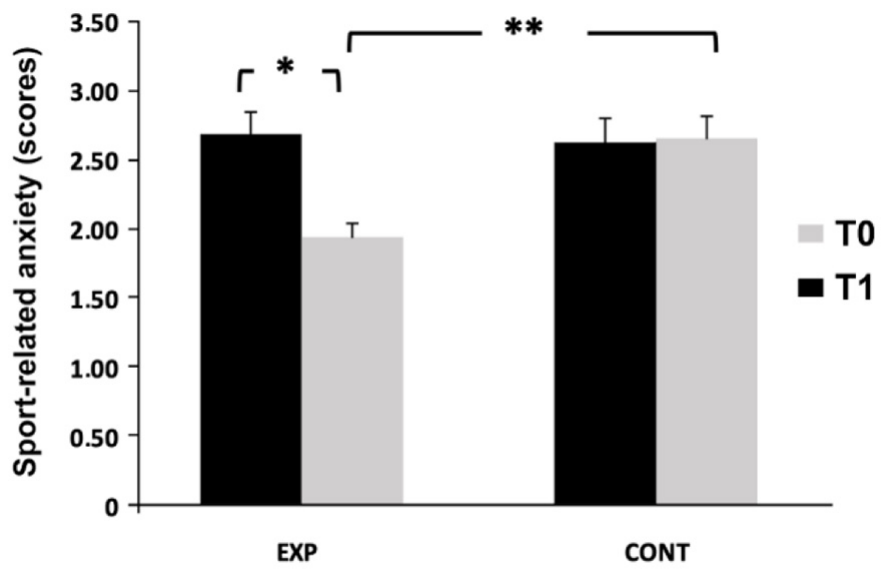
Sport-related anxiety (SAS-2) measured before (T0) and after the intervention (T1) in the control (CONT) and experimental (EXP) groups. *p < 0.05; **p < 0.001, Bonferroni post hoc tests.

Mixed ANOVA revealed significant main effects of time, *F*_(1, 62)_ = 10.91, *p* = 0.0016, η^2^*?* = 0.15, and a significant group × time interaction, *F*_(1, 62)_ = 12.89, *p* = 0.0007, η^2^*?* = 0.17, with no significant main effect of group, *F*_(1, 62)_ = 2.67, *p* = 0.107. *Post hoc* comparisons indicated a significant reduction in anxiety in the experimental group (*p* < 0.001, *d* = 0.95), with no change in controls. At posttest, anxiety scores were significantly lower in the experimental group (*p* < 0.05, *d* = 0.86). Mean anxiety decreased from 2.69 ± 0.93 to 1.94 ± 0.62 in the experimental group and remained stable in controls (2.63 ± 1.04 vs. 2.66 ± 1.00; Figure 4).

### 3.4 Basketball performance

Passing: Pass Accuracy improved from 2.22 ± 0.79 to 2.94 ± 1.11, and Pass Fail decreased from 7.78 ± 0.79 to 7.00 ± 1.21 in the experimental group, with no significant change in controls. ANOVA showed significant main effects of Time and Group × Time interactions (Pass Accuracy: *F* = 6.85, *p* = 0.011 η^2^*p* = 0.1; Pass Fail: *F* = 6.93, *p* < 0.05; η^2^*p* = 0.101; Table 2).

**Table 2:** Passing and shooting performance scores (mean ± standard deviation).

Performance	Exp		CONT		ANOVA		
	T0	T1	T0	T1	Time × group (*p*)	Time (*p*)	group (*p*)
Pass accuracy	2.21 ± 0.79	2.93 ± 1.11*^&^	2.25 ± 0.88	2.21 ± 0.79	0.01	0.02	–
Pass fail	7.78 ± 0.79	7 ± 1.21*^&^	7.75 ± 0.87	7.78 ± 0.79	0.01	0.02	0.04
Shot made	0.90 ± 0.89	1.46 ± 0.76^**^	1.5 ± 1.13	1.4 ± 0.94	< 0.001	0.014	–
Shot miss	9.09 ± 0.89	8.56 ± 0.8^**^	8.5 ± 1.13	8.59 ± 0.94	0.002	0.026	–

Exp, Experimental group; CONT, control group; T0, pretest; T1, posttest; *P* values adjusted for multiple testing by Bonferroni; ^*^, ^**^Significant difference compared with T0 (*p* < 0.01 and *p* < 0.001, respectively); ^&^Significant difference groups at T1 with *p* < 0.05.

These findings suggest that the adapted intervention led to a significant improvement in overall passing performance, with participants in the experimental group demonstrating both higher accuracy and fewer failed passes, while the control group remained stable.

Shooting: Shot Made increased from 0.90 ± 0.89 to 1.46 ± 0.76 and Shot Miss decreased from 9.09 ± 0.89 to 8.56 ± 0.80 in the experimental group, while controls remained stable. Significant Time × Group interactions were observed for both metrics (Shot Made: *F* = 12.35, *p* < 0.001 η^2^*p* = 0.166; Shot Miss: *F* = 10.56, *p* = 0.002 η^2^*p* = 0.146). Detailed scores are presented in Table 2.

### 3.5 Correlation analysis of psychological and performance change scores

The analysis of change scores revealed a strong positive association between reductions in sport anxiety (ΔSAS) and emotional eating (ΔDEBQ; *r* = 0.52, *p* < 0.01), indicating that participants who experienced a greater decrease in anxiety also tended to show a parallel decline in emotion-driven eating behaviors.

**Figure 5:**
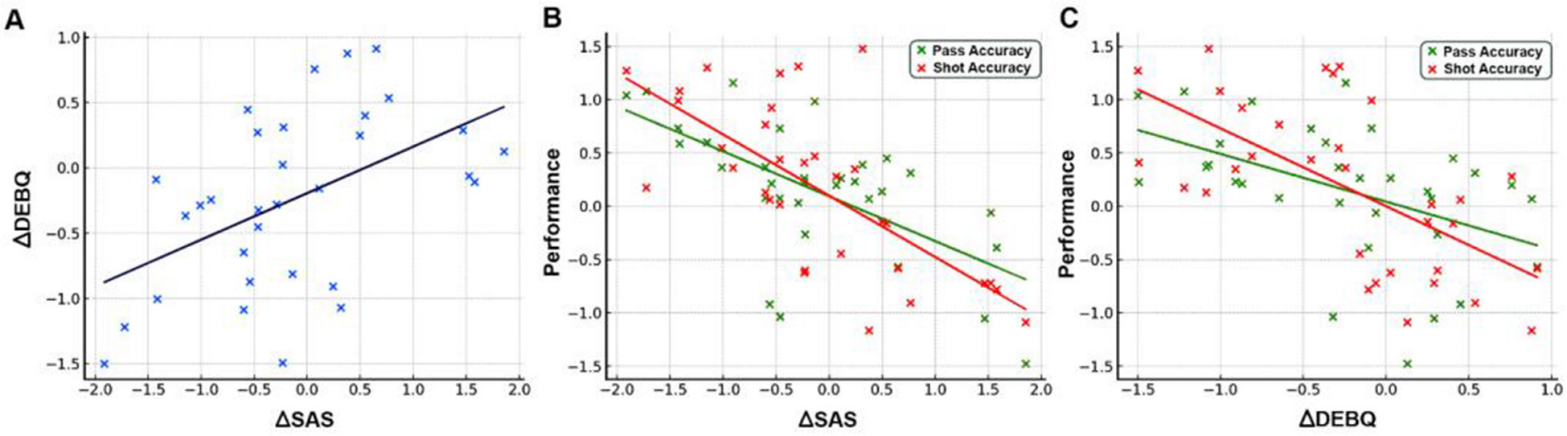
Correlations between changes in psychological measures and basketball performance. **(A)** ΔSAS vs. ΔDEBQ (*r* = 0.52, *p* < 0.01). **(B)** ΔSAS vs. pass accuracy (*r* = −0.38, *p* < 0.01) and shot accuracy (*r* = −0.41, *p* < 0.01). **(C)** ΔDEBQ vs. pass accuracy (*r* = −0.31, *p* < 0.01) and shot accuracy (*r* = −0.35, *p* < 0.01).

Furthermore, improvements in both psychological indicators were significantly related to enhanced basketball performance. Specifically, greater reductions in sport anxiety and emotional eating were associated with higher gains in pass accuracy (ΔSAS: *r* = −0.38; ΔDEBQ: *r* = −0.31; both *p* < 0.01) and shot accuracy (ΔSAS: *r* = −0.41; ΔDEBQ: *r* = −0.35; both *p* < 0.01; Figure 5).

These results indicate that participants with the greatest reductions in anxiety and emotional eating also showed the most improvement in basketball performance, highlighting the link between emotional regulation and motor skill optimization.

## 4 Discussion

Adolescence represents a critical window for the prevention and management of obesity, particularly in girls who are highly vulnerable to both physical and psychological consequences. The present study demonstrates that tailoring basketball sessions to the specific needs of adolescent girls with moderate obesity can produce meaningful improvements in BMI, emotional eating, sport-related anxiety, and technical performance. By integrating pedagogical adaptations into physical education, our findings highlight how structured, supportive, and engaging interventions can not only enhance physical outcomes but also foster emotional wellbeing and confidence, offering a holistic approach to adolescent health.

BMI significantly decreased in the experimental group, whereas no change was observed in the control group. This aligns with prior studies (Samsury et al., 2025; Schneiders et al., 2021), which demonstrated that structured, progressive, and mastery-oriented physical activity programs lead to measurable reductions in adipose tissue among adolescents with obesity. Several mechanisms may explain this improvement: Alternating phases of moderate-to-high intensity with active recovery likely increased energy expenditure without causing overload. This process stimulates metabolic lipolysis while minimizing cortisol peaks that favor visceral fat storage (Samsury et al., 2025). Additionally, reducing competitive pressure and fostering a supportive motivational climate may have decreased hypothalamic–pituitary–adrenal (HPA) axis reactivity, indirectly contributing to hormonal balance (van Paridon et al., 2017). Finally, progressive consolidation of motor skills through didactic adaptations (e.g., limited defensive constraints, mandatory substitutions) improved motor efficiency and energy economy, further explaining BMI reduction over the intervention period (Slimi et al., 2025a).

Emotional eating scores markedly decreased in the experimental group, whereas a slight increase was observed in the control group. These findings support previous evidence that physical practice in an emotionally secure context can regulate impulsive eating behaviors (Alexatou et al., 2025; Sun et al., 2024). In the experimental program, combining physical effort, motor success, and positive social interactions promoted endorphin and dopamine release. This process supports emotional stability and reduces reliance on food as a reward (Zhu, 2024). The supportive peer and teacher climate emphasizing individual progress without social judgment, may contribute to improved stress regulation and emotion-driven eating through mechanisms suggested in previous neurophysiological studies, though these effects remain hypothetical in the absence of direct biological measurements (Biggs et al., 2014; Sahi and Eisenberger, 2023).

Furthermore, progressive mastery of technical tasks and increased perceived control likely enhanced self-esteem and psychological satisfaction, replacing compensatory eating with intrinsic reinforcement derived from motor mastery and the pleasure of movement (Flack et al., 2023).

Sport-related anxiety also significantly decreased in the experimental group. This aligns with previous findings (Yu et al., 2024; Anker et al., 2024), highlighting that reducing competitive pressure and fostering an autonomous, supportive learning environment attenuates anxiety in at-risk adolescents. In our study, the pedagogical progression—from simple 1-on-1 drills to collective 5-on-5 games with mandatory rotations—enhanced perceived competence and created an emotionally protective climate. Neurophysiologically, this reduction in anxiety is consistent with prior literature suggesting potential involvement of the limbic system and dorsolateral prefrontal cortex in cognitive and emotional regulation, as well as modulation of serotonin and norepinephrine activity; however, these mechanisms remain speculative in the absence of direct biological measures (Warren et al., 2020; Ren and Xiao, 2023). Moreover, the cooperative social learning dynamic likely reduced fear of judgment and failure, decreasing anxiety vigilance and avoidance behaviors commonly observed in adolescent girls with obesity during sports (Ayala et al., 2021; Spruit et al., 2016).

Regarding technical performance, the experimental group showed significant improvements in passing and shooting accuracy, who demonstrated that motor learning in psychologically safe environments enhances the consolidation of perceptual–motor schemas and action efficiency. These improvements may result from functional reorganization of motor networks through contextualized repetition and intrinsic feedback. The program's progressive structure—from individual drills to simplified collective situations—facilitated gradual automation of motor control and optimized intersegmental coordination (Krakauer and Mazzoni, 2011; Wulf and Shea, 2002).

Reduced anxiety likely freed attentional resources, improving motor planning and execution (Eysenck et al., 2007; Wulf and Lewthwaite, 2016).

Additionally, combined social feedback and self-assessment may have strengthened perception–action coupling, leading to finer movement calibration and better anticipation of trajectories—skills essential for basketball success (Williams et al., 2010; Araújo and Davids, 2006).

Correlations between decreased anxiety, reduced emotional eating, and improved technical performance highlight the interdependence of psychological and motor domains. Neurocognitively, these relationships may be mediated by shared circuits involved in motivation, emotional regulation, and motor control, particularly mesolimbic dopaminergic pathways and the anterior cingulate cortex (Asemi et al., 2015; Lin et al., 2020; Albert et al., 2012).

Reduced anxiety lowers cognitive load, optimizing attentional resources for motor tasks, while improved emotional regulation enhances intrinsic motivation and persistence (Eysenck et al., 2007; Deci, 2000).

Higher perceived competence and stronger social cohesion further increase psychological satisfaction, generating positive feedback loops that reinforce both motor engagement and emotional stability (Ntoumanis, 2009; Ryan, 2020).

From a practical perspective, teachers and PE practitioners can implement these adaptations by structuring sessions progressively—from individual drills to collective play—while adjusting difficulty based on students' skill levels. Limiting defensive constraints, using mandatory rotations, and fostering a supportive, non-judgmental learning climate are recommended to maintain motivation and engagement. Practitioners should monitor student participation and ensure session consistency, providing sufficient opportunities for motor mastery while reducing competitive pressure. These pedagogical strategies may help teachers and coaches create inclusive learning environments that support both psychological wellbeing and motor skill development in adolescent girls with obesity.

Nevertheless, this study has some limitations. First, the study was conducted on a single sample of 64 adolescent girls from one school within a specific cultural and educational context. This limits the generalizability of the findings, as differences in gender, age groups, school systems, and cultural or socioeconomic backgrounds may influence both engagement in physical activity and responsiveness to didactic adaptations. In addition, the lack of objective monitoring of physical activity outside the sessions and dietary intake limits the causal interpretation of changes in BMI and emotional eating. Finally, the study did not include direct physiological or neurobiological measures (e.g., cortisol levels, brain imaging), which constrains understanding of the underlying mechanisms driving emotional and motor regulation.

Future research should replicate this intervention across multiple schools, regions, and diverse cultural settings to test the robustness, reproducibility, and universality of the observed effects, and to determine whether similar improvements in BMI, emotional regulation, anxiety, and technical performance occur in broader adolescent populations. Including biomarkers and neurophysiological assessments, such as stress hormones and functional brain imaging, would clarify mechanisms underlying emotional and motor adaptations. Additionally, testing similar interventions in other team or mixed sports could provide insights for designing inclusive, evidence-based programs in physical education. Future studies should also explore the impact of intervention duration and session frequency on long-term outcomes to identify optimal program design for sustained improvements in BMI, emotional regulation, anxiety, and motor performance.

## 5 Conclusions

This study demonstrates that didactically adapted basketball sessions in physical education can produce meaningful improvements in BMI, emotional eating, sport-related anxiety, and technical performance among adolescent girls with obesity. Tailoring activities to the participants' abilities, providing a supportive and non-judgmental environment, and progressively structuring tasks from individual drills to collective play appear to enhance both psychological wellbeing and motor skill development. These findings highlight the potential of pedagogically informed interventions to promote holistic health in vulnerable adolescent populations. Future research should explore the long-term sustainability of these effects, include objective physiological and neurobiological measures, and examine the applicability of similar adaptations in other sports contexts to inform inclusive, evidence-based physical education practices.

## Data Availability

The raw data supporting the conclusions of this article will be made available by the authors, without undue reservation.
