# Correction: Didactic adaptation of basketball in physical education to reduce anxiety and emotional eating in adolescent girls with obesity: a randomized controlled trial

**DOI:** 10.3389/fpsyg.2026.1856920

**Published:** 2026-04-28

**Authors:** Oumayma Slimi, Amayra Tannoubi, Raouf Nasri, Mohamed Abdelkader Souissi, Santo Marsigliante, Antonella Muscella, Jolita Vveinhardt

**Affiliations:** 1 High Institute of Sport and Physical Education, University of Sfax, Sfax, Tunisia; 2 Research Laboratory: “Education, Motricité, Sport et Santé”, EM2S, LR19JS01, High Institute of Sport and Physical Education of Sfax, University of Sfax, Sfax, Tunisia; 3 Department of Education, High Institute of Sport, and Physical Education of Gafsa, University of Gafsa, Gafsa, Tunisia; 4 Sports Performance Optimization Research Laboratory (LR09SEP01), National Center for Sports Medicine and Science (CNMSS), Tunis, Tunisia; 5 Physical Activity, Sport and Health Research Unit, UR18JS01, National Observatory of Sport, Tunis, Tunisia; 6 Department of Biological and Environmental Sciences and Technologies (DiSTeBA), University of Salento, Lecce, Italy; 7 Klaipėdos valstybinė kolegija/Higher Education Institution, Klaipėda, Lithuania

**Keywords:** adapted physical education, adolescent girls, basketball training, emotional eating, motor performance, obesity, sport-related anxiety

Affiliation “Klaipėdos valstybinė kolegija/Higher Education Institution, Klaipėda, Lithuania” was omitted for author Jolita Vveinhardt. This affiliation has now been added for author Jolita Vveinhardt.

Author “Jolita Vveinhardt” was erroneously assigned to affiliation Vytautas Kavolis Interdisciplinary Research Institute, Vytautas Magnus University, Kaunas, Lithuania. This affiliation has now been removed for author Jolita Vveinhardt.

The original version of this article has been updated.

